# Reliable fabrication of transparent conducting films by cascade centrifugation and Langmuir–Blodgett deposition of electrochemically exfoliated graphene

**DOI:** 10.3762/bjnano.13.58

**Published:** 2022-07-18

**Authors:** Teodora Vićentić, Stevan Andrić, Vladimir Rajić, Marko Spasenović

**Affiliations:** 1 Center for Microelectronic Technologies, Institute of Chemistry, Technology and Metallurgy, National Institute of the Republic of Serbia, University of Belgrade, Njegoševa 12, Belgrade 11000, Serbia; 2 INS Vinča, Department of Atomic Physics, University of Belgrade, Mike Petrovića Alasa 12–14, 11351 Belgrade, Serbia

**Keywords:** 2D materials, cascade centrifugation, graphene, Langmuir–Blodgett deposition, transparent conductors

## Abstract

Electrochemical exfoliation is an efficient and scalable method to obtain liquid-phase graphene. Graphene in solution, obtained through electrochemical exfoliation or other methods, is typically polydisperse, containing particles of various sizes, which is not optimal for applications. We employed cascade centrifugation to select specific particle sizes in solution and prepared thin films from those graphene particles using the Langmuir–Blodgett assembly. Employing centrifugation speeds of 3, 4, and 5 krpm, further diluting the solutions in different volumes of solvent, we reliably and consistently obtained films of tunable thickness. We show that there is a limit to how thin these films can be, which is imposed by the percolation threshold. The percolation threshold is quantitatively compared to results found in literature that are obtained using other, more complex graphene film fabrication methods, and is found to occur with a percolation exponent and percolative figure of merit that are of the same order as results in literature. A maximum optical transparency of 82.4% at a wavelength of 660 nm is obtained for these films, which is in agreement with earlier works on Langmuir–Blodgett assembled ultrasonic-assisted liquid-phase exfoliated graphene. Our work demonstrates that films that are in all respects on par with films of graphene obtained through other solution-based processes can be produced from inexpensive and widely available centrifugal post-processing of existing commercially available solutions of electrochemically exfoliated graphene. The demonstrated methodology will lower the entry barriers for new research and industrial uses, since it allows researchers with no exfoliation experience to make use of widely available graphene materials.

## Introduction

The interest in graphene and other 2D materials keeps growing, especially since the initial delve into fundamental properties was augmented with an outlook towards potential applications [1]. Over the past decades, a great number of different methods for the synthesis of graphene and other 2D materials has been proposed, including micromechanical cleavage [2], chemical vapor deposition (CVD) [3–7], epitaxial growth on different substrates [8–9], and the chemical reduction of graphene oxide (GO) [10–11]. In 2008, production of graphene by liquid-phase exfoliation (LPE) of graphite through sonication of graphite powder in *N*-methylpyrrolidone (NMP) was first proposed by Coleman et al. [12] as a synthesis method with high potential for scaling. Since then, LPE has developed into a common, highly scalable method for graphene synthesis in liquid media. This method is used for the production of 2D nanosheets with lateral sizes ranging from 100 nm to 100 µm and thicknesses in the range of 1–10 layers, in a range of different liquids, at a wide range of concentrations [13–14].

The mechanism of ultrasonic exfoliation involves ultrasonic waves in liquid media creating bubbles or voids in the liquid, which generate shear forces or cavitation bubbles upon collapsing, which then break up the bulk 2D materials into mono- and few-layer nanosheets [15–16]. The choice of solvent for LPE is made based on surface energy considerations, compatible solvents include NMP, dimethylformamide (DMF), *N*,*N*-dimethylacetamide (DMA), γ-butyrolactone (GBL), 1,3-dimethyl-2-imidazolidinone (DMEU), and *ortho*-dichlorobenzene (*o*-DCB) [12,17–18]. Exfoliation in NMP has led to minimally oxidized graphene sheets with approximately 28% monolayer flakes, and more than 75% of sheets with a thickness of less than six layers [12].

An alternative to LPE that has subsequently been developed is electrochemical exfoliation, whereby graphene is exfoliated in an electrolyte from an electrode made of graphite [19]. In electrochemical exfoliation, ions from the electrolyte flow towards the graphite electrode and intercalate between the graphene layers. The electrochemical reaction provides a driving force to break van der Waals forces, leading to exfoliation [20]. Electrochemical exfoliation offers an alternative to LPE that is both scalable and widely available. It has been used to make graphene for various applications, including energy storage [21–22].

Both ultrasound-assisted LPE and electrochemical exfoliation result in solutions that contain flakes of different sizes, that is, the solutions are polydisperse. Polydispersity is a significant problem regarding the use of solution-processed graphene, because many applications are size-dependent. On the one hand, for example, for use in composites, flakes with lateral sizes larger than 1 µm are preferred [23]. On the other hand, thinner (thus also laterally smaller [24]) flakes have a higher transparency, with potential use in transparent conductors. Size selection of 2D material flakes in solution has thus become a key challenge for the practical use of solution-processed 2D materials [24–25].

The flake size can either be controlled during exfoliation or selected after exfoliation. Processing parameters that control flake size during exfoliation include the choice and concentration of solvent [25], a process control alternating sonication with shear mixing [26], or the duration of exfoliation [27]. Using LPE for 2D materials that are size-selected during exfoliation limits their use to research groups with expertise in this method. After exfoliation, sizes can be selected by centrifugal processing, which narrows the nanosheet size and thickness distribution, depending on the centrifugation parameters. However, it is important to consider the impact of buoyant density and drag coefficient of the materials, as well as the viscosity of the solvent and many other parameters to achieve the desired results [28]. It was demonstrated by Coleman et al. that controlled centrifugation can be used for the selection of liquid-phase exfoliated graphene dispersions with mean flake sizes in the range from 1 to 3.5 µm [29]. Since centrifugation is a much more facile process than exfoliation, and centrifuges are widely available, post-exfoliation size selection is the route to take towards the mass use of 2D materials in solution.

Although size selection through post-processing with cascade centrifugation has been demonstrated in the context of ultrasonic LPE materials [30–31], to the best of our knowledge, the method has not been applied to electrochemically exfoliated graphene, nor have thin films made from dispersions following size selection through centrifugation been studied for their optoelectronic properties. Here, we present size selection through cascade centrifugation of commercially obtained electrochemically exfoliated graphene. We follow the Langmuir–Blodgett (LB) method to deposit graphene flakes from solution as uniform thin films. LB has proven to be a method that yields reliable graphene films that have been used as transparent conductors [27,32–34] and gas sensors [35–36]. By measuring optical transmittance and electrical resistance of the deposited films, we find a tradeoff between transparency and electrical performance for applications in transparent conductors. We demonstrate that, below a certain critical thickness, graphene films deposited with LB reach a percolation limit, which imposes a minimum achievable film thickness for a reasonable electrical conductivity. For both, our films made from electrochemically exfoliated graphene and literature-referenced films made from ultrasonic LPE graphene, the percolation limit is reached at an optical transmittance of ca. 83%. This number quantifies the maximum transmittance achievable with LB assembly of graphene films made from solution-dispersed material, for a reasonable electrical conductivity. Comparisons of our obtained percolative figure of merit and percolation exponent with those observed in literature reveal that the quality of the films obtained with our demonstrated method is on par with graphene films made with other methods that make use of liquid-phase graphene. Hence, we demonstrate that commercially obtained solutions of graphene can be post-processed with a simple laboratory centrifuge and deposited into thin films with a quality on par with films obtained with other methods that require more effort from the lab workers, as well as exfoliation expertise and equipment.

## Experimental

### Cascade centrifugation

**Figure 1:**
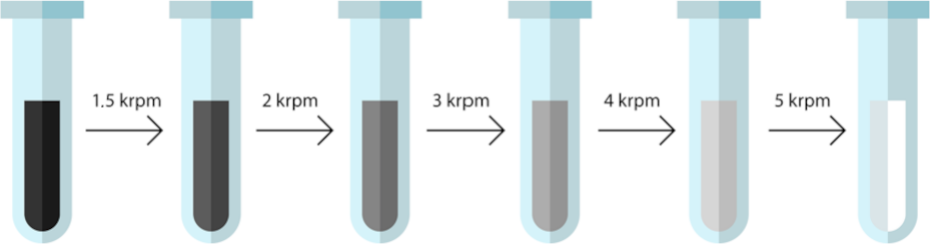
The process of cascade centrifugation.

In order to achieve homogeneous films with defined particle sizes, a dispersion of electrochemically exfoliated graphene from Sixonia Tech GmbH (G-DI5P-NMP-C50-2+, Dresden, Germany) was processed by cascade centrifugation (centrifuge model: COLO LACE16 from Novo Mesto, Slovenia, rotor R30403 with radius 8.19 cm). The commercially obtained solution contained a dispersion of graphene in NMP. Although many solvents are commercially available, NMP was the solvent of choice because of its favorable properties regarding LB deposition [14]. 1 mL of dispersion was initially centrifuged at a rate of 1500 rpm (relative centrifugal force, RCF, equal to 206*g*). The obtained centrifugation sediment contained the largest nanosheets of the initial dispersion. The supernatant was subsequently centrifuged at higher rates: 2, 3, 4, and 5 krpm (366*g*, 824*g*, 1465*g*, and 2289*g*). At each step of centrifugation, the sediments with slightly smaller graphene sheets were gathered and the supernatants were used for the following cascade step. The sediments were collected and redispersed in a specific volume of NMP (Sigma-Aldrich M79603), which ranged between 250 and 1000 µL. This method is schematically described in Figure 1.

### Film deposition

**Figure 2:**
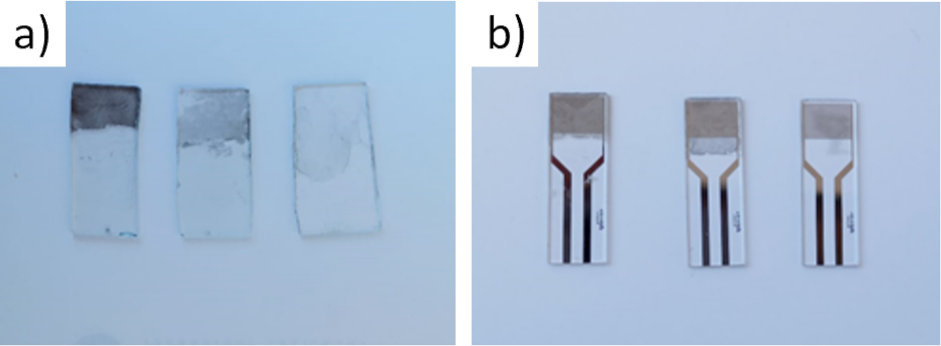
Deposited graphene films at centrifugation rates of 3, 4, and 5 krpm (824*g*, 1465*g*, and 2289*g*, respectively) from left to right; (a) glass substrate, (b) Metrohm DropSens substrates.

In order to study the optical properties of the produced graphene films, graphene from solution was deposited onto glass substrates with dimensions of ca. 2 cm × 1 cm (Figure 2a). Although a great number of different types of substrate materials have been used, such as silica [37], chromium [38], tin [39], silver [40], or platinum [41], glass substrates were used in this paper. Glass is generally a popular choice, not just because glass slides are inexpensive and widely available, but also because the optical characteristics of deposited films can be subsequently examined [42]. For measuring the electrical resistance, the film was deposited onto Metrohm DropSens substrates with a pair of interdigitated electrodes (G-IDEPT10, Oviedo, Spain, Figure 2b). Using an automated pipette, the entire specific volume of the graphene dispersion in NMP was vertically dripped onto the surface of deionized water. Because the volumes ranged from 250 to 1000 µL, while the amount of graphene in that volume was kept fixed, the concentration of graphene was varied. As the film of graphene formed on the surface of the water, the LB method was used to deposit the film onto the target substrate [32].

### Film characterization

To study optical properties of the fabricated samples, UV–vis spectroscopy was performed (Thermo Fisher Scientific EVO 60, Madison, USA). A xenon lamp was used as a light source. The glass samples were mounted on a holder and inserted into the light path. Optical transmittance was measured by subtracting the signal from a baseline reference signal obtained when a clean glass substrate without graphene was inserted into the light path.

Graphene film resistance was measured by inserting the substrates with electrodes into an electrode connector (DRP-CACIDE, Metrohm, Oviedo, Spain) and the acquiring resistance with a handheld digital multimeter.

Optical dark-field microscopy of the films was performed with a magnification of 10× (Olympus BX53M). Scanning electron microscopy (SEM) was performed with a FESEM (FEI Scios 2, Thermo Fisher Scientific, Waltham, MA, USA) at a chamber pressure of 1 × 10^−4^ Pa with electron beam voltages set between 1 and 30 kV, depending on the film. Films that are shown in optical dark-field microscopy and SEM have been made from solutions that have been diluted with 500 µL of NMP.

## Results

### Optical observation

**Figure 3:**
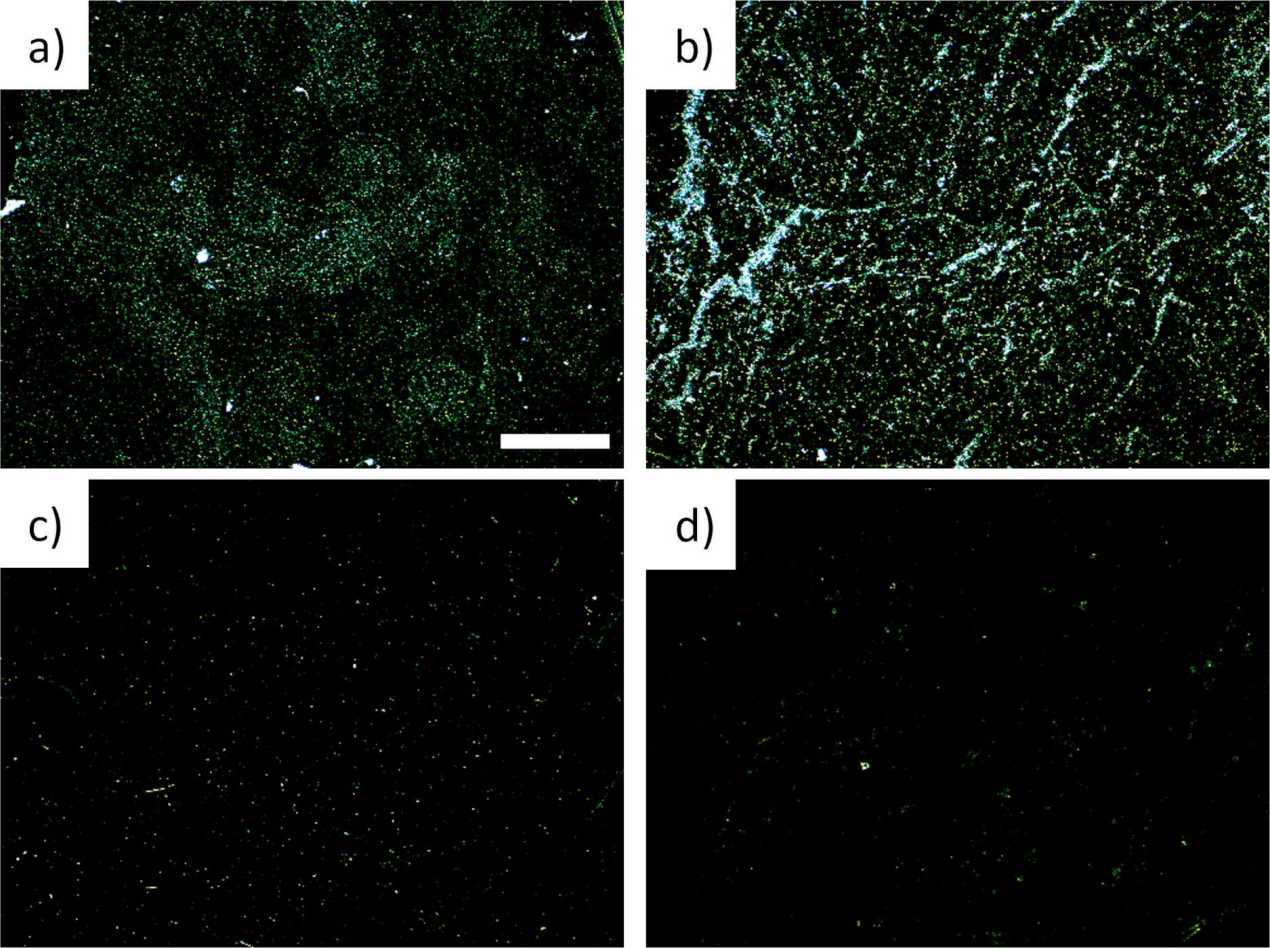
Dark-field microscopic images of films produced from (a) the initial graphene dispersion and from films produced after centrifugation at (b) 3 krpm (824*g*), (c) 4 krpm (1465*g*), and (d) 5 krpm (2289*g*). The scale bar in panel (a) is 200 µm long and is valid for all panels. All images show an area of 1.15 × 0.86 mm.

Films deposited from solutions obtained from different centrifugation protocols are expected to have different thicknesses, due to the different size of the flakes in the solutions. A visual inspection of the images in Figure 2 confirms that slower centrifugation rates (samples on the left) yield thicker films than faster centrifugation rates (samples on the right), which is expected because flakes are thicker and laterally larger when processed at slower rates. To gain further insight into the thickness and quality of these films, we performed optical dark-field microscopy with a magnification of 10×. Photographs of deposited graphene films from solutions processed at different centrifugation rates are depicted in Figure 3. Films made directly from the initial polydisperse solution (Figure 3a) show regions with a high density of scattering centers (green and white points) intermixed with regions with a lower density of scattering centers (darker regions with few bright points). Size-selected solutions yield films that are more uniform, with dark-field microscopy revealing homogeneous distributions of scattering centers. Hence, we find that films from polydisperse films are not as homogeneous as films made from solutions that have been processed by centrifugation for size selection. There is an evident decrease in the density and intensity of scattering centers with increasing centrifugation rate, which indicates films with a decreased density of large particles.

We quantify the number of scattering centers by analyzing the dark-field images using the software imageJ. First, we set the brightness threshold for what counts as a scattering particle. As these are dark-field images, anything that is not completely dark is counted as a scattering particle. Thus, we set the threshold at a brightness value of 3 (on a scale from 0 to 255). Such an analysis yields results that are consistent with intuitive observation (Table 1).

**Table 1:** Analysis of scattering centers from dark-field observation. Both the number of particles and their total area decreases with centrifugation rate.

Centrifugation rate	Particle count	Total area	% area

3 krpm (824*g*)	5001	58972	32.329
4 krpm (1465*g*)	1909	3029	1.661
5 krpm (2289*g*)	1053	2159	1.184

**Figure 4:**
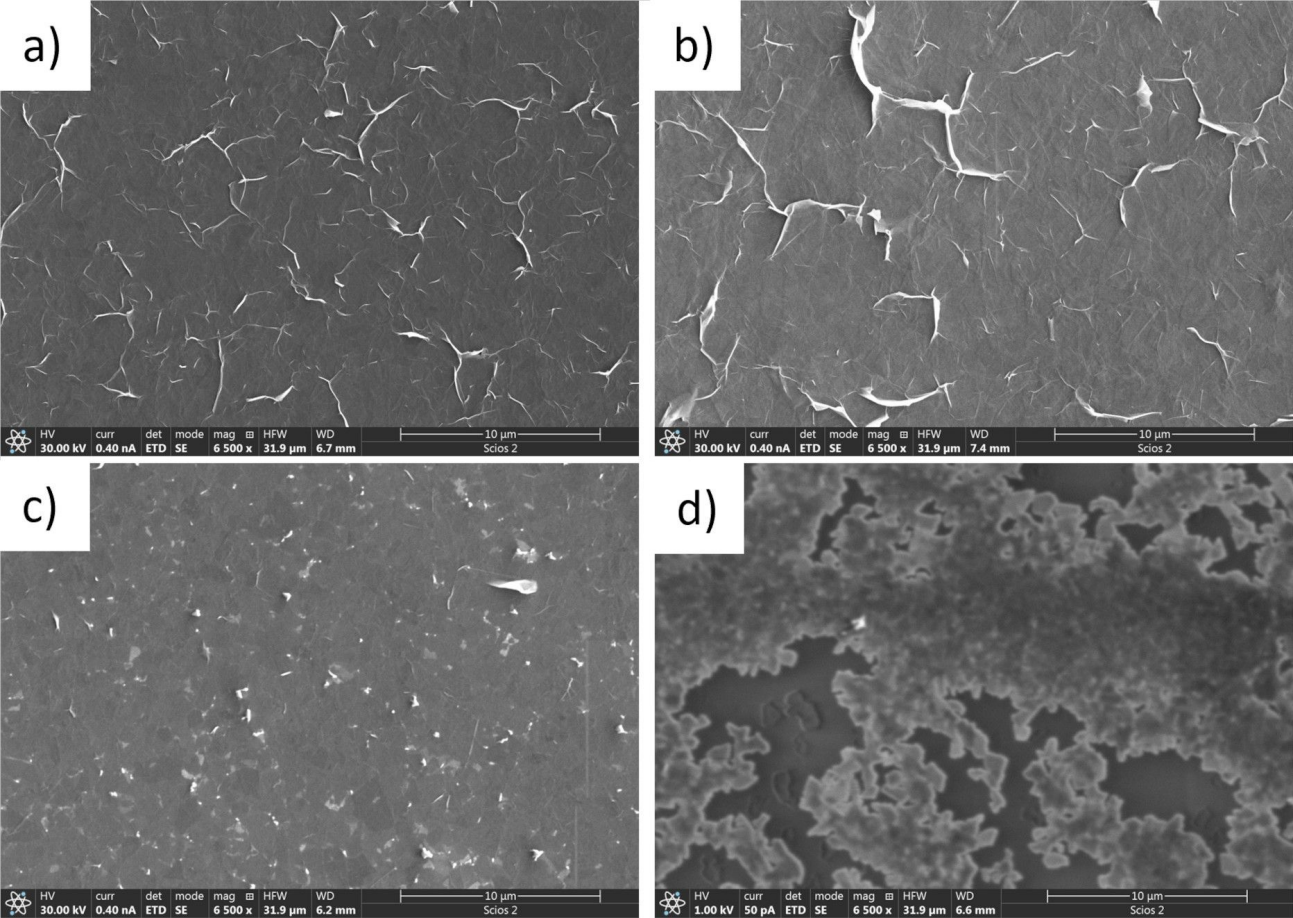
Scanning electron micrographs of films produced from the (a) initial graphene dispersion and from films produced after centrifugation at (b) 3 krpm (824*g*), (c) 4 krpm (1465*g*), and (d) 5 krpm (2289*g*).

The film structure was further investigated with SEM, shown in Figure 4. In general, the films look alike when they are made of the as-purchased uncentrifuged solution and when they are made from solutions centrifuged at 3 krpm (824*g*) and at 4 krpm (1465*g*). In these three cases, SEM reveals continuous graphene films. The films contain some wrinkles or flake edges, showing up as bright lines in SEM. Upon closer inspection, the contrast varies slightly across the films, which is likely due to local thickness variations. Overall, the films resemble those made earlier with ultrasonic liquid-phase exfoliation followed by LB deposition [14]. However, the film made from a solution that has been centrifuged at 5 krpm (2289*g*) is strikingly different from the ones made with lower centrifugation speeds. The 5 krpm (2289*g*) film has an irregular structure, resembling a fractal coastline. It is also noted that the conductivity of this film is much lower than those of the other three films, as SEM operation quickly leads to surface charging effects.

### Optoelectronic properties

**Figure 5:**
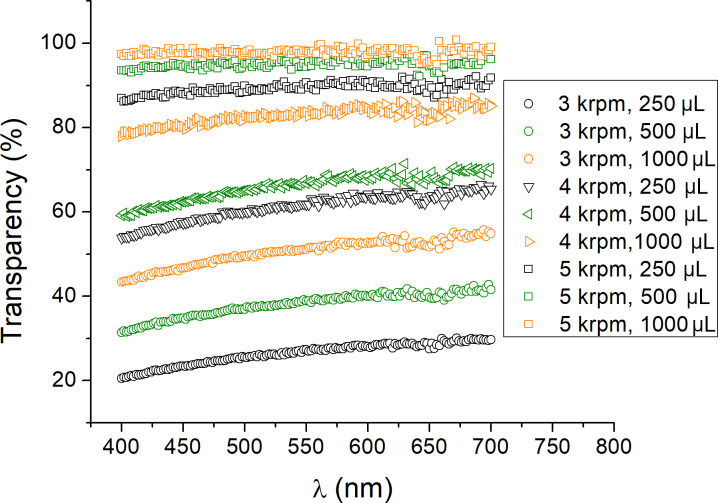
UV–vis spectra of deposited graphene films at different centrifugation rates, redispersed in specified volumes of NMP.

UV–vis spectra of the deposited graphene films at different centrifugation rates, redispersed in specified volumes of NMP, are given in Figure 5. The optical transmission spectra are expectedly uniform across the visible part of the spectrum. It is evident that the optical transparency can be controlled by the centrifugation rate, as well as by tuning the concentration of graphene particles by redispersing in different volumes of NMP. Optical transparencies were measured at the wavelength of 660 nm and the number of graphene layers was calculated for each sample, taking into account an absorption of 2.3% for each layer of graphene, as in the work by Bonaccorso and co-workers [43].

Although atomic force microscopy (AFM) is often employed to characterize graphene films [2,12,14,44], applying that method to films that consist of heterogeneous flakes, such as Langmuir–Blodgett-deposited films, is more difficult. Since the thickness varies from flake to flake, only an average film thickness over a certain area makes sense. The area over which average thickness can be measured with AFM is limited by the scan size, at a maximum of about 50 µm × 50 µm. The best method for measuring the average film thickness with AFM is to make scans that show the underlying substrate as well as the film itself and to make a histogram of measured heights, where a narrow peak related to the substrate and a broader peak related to the film surface appear. Then, the thickness is measured as the distance between those two peaks in the histogram [14,33]. With that method, the accuracy of measuring the average film thickness depends on the size of the scanned area. Optical transparency, in contrast, is a good measure of film thickness averaged over the size of the optical spot. For example, in [33], it was shown that thickness measurements from optical transmittance match the results obtained with AFM, for continuous films. However, for films that do not cover the substrate completely, such as the one shown in Figure 4d, measuring the average film thickness with AFM would be impossible, because one needs to measure over a large area to take into account regions with flakes as well as regions without. Optical transmittance is a good measure of the average thickness even in the case of films that do not cover the substrate completely.

**Figure 6:**
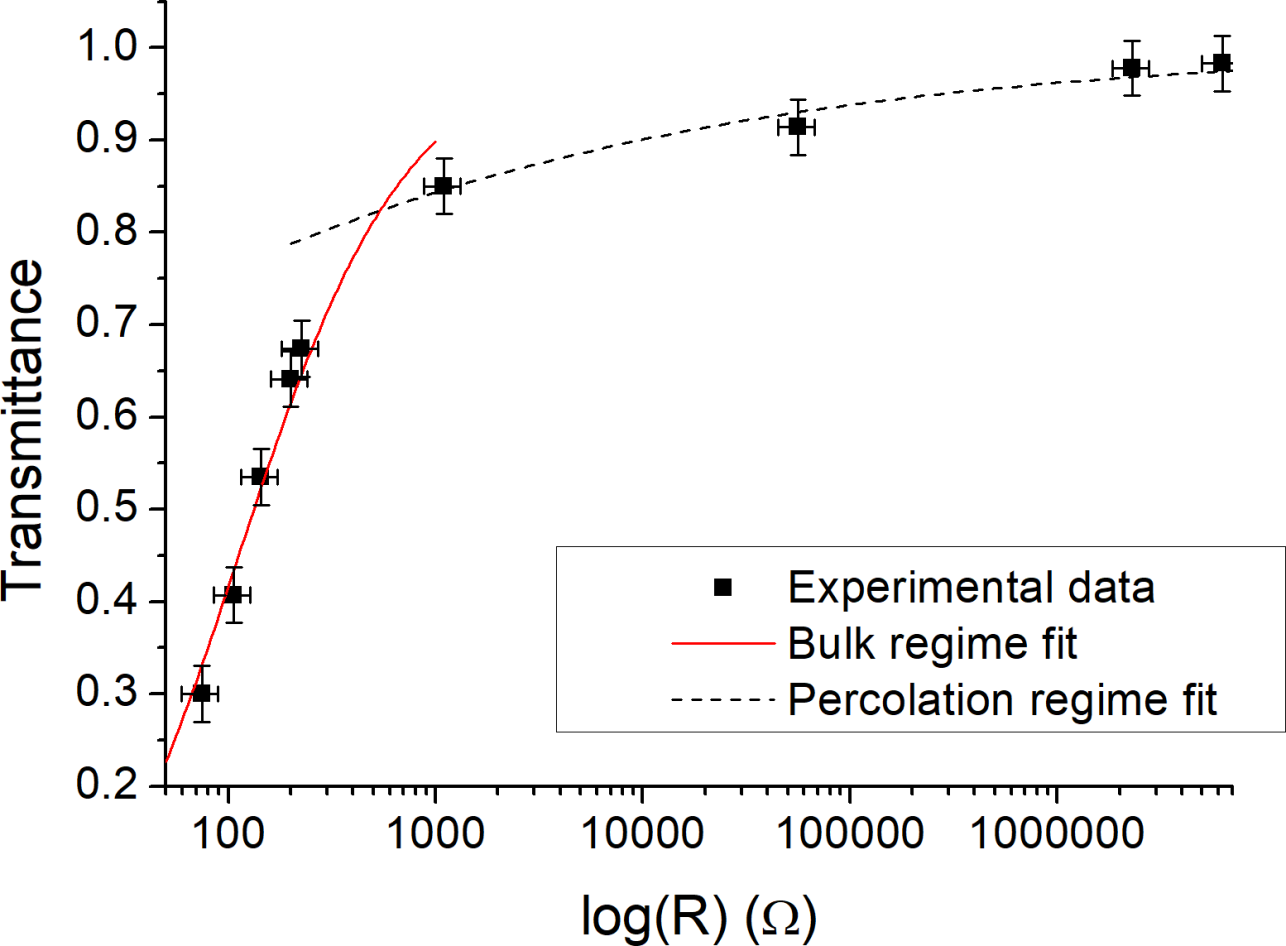
Dependence of the optical transparency on the electrical resistance of graphene films on a semi-logarithmic scale. The data can be divided into two regimes, the bulk regime (solid red line fit to Equation 1) and the percolation regime (dashed black line fit to Equation 2).

To analyze the electrical properties of the synthesized graphene films, resistance values of each film were measured. Resistance values, optical transparency values, and the average number of layers in each graphene film are shown in Table 2. A larger number of graphene layers implies a smaller electrical resistance. The relation of optical transparency and electrical resistance for all samples is given in Figure 6. The measured optical transmittance varied slightly depending on the exact spot chosen on each given sample. The measured resistance varied by about 20% between different samples prepared with identical methodology. Since only several samples were made for each set of processing parameters, the error bars in Figure 6 represent the maximum deviation from the mean observed during experimentation.

**Table 2:** Measured optical transparency, number of layers, and electrical resistance for samples obtained at different centrifugation rates, dissolved in the specified volumes of NMP.

	*V* (NMP)	Optical transparency	Average number of layers	Resistance

3 krpm (824*g*)	250 µL	30.0%	30.4	74.6 Ω
	500 µL	40.7%	25.8	106.4 Ω
	1000 µL	54.0%	20.0	144.0 Ω
4 krpm (1465*g*)	250 µL	64.1%	15.6	200.0 Ω
	500 µL	67.4%	14.2	226.0 Ω
	1000 µL	85.0%	6.5	1.1 kΩ
5 krpm (2289*g*)	250 µL	91.4%	3.7	56.1 kΩ
	500 µL	94.0%	2.3	2.3 MΩ
	1000 µL	98.2%	0.8	6.3 MΩ

## Discussion

The images in Figure 2 show that the thickness of the deposited graphene films decreases with increasing centrifugation rates, while Figure 3 indicates that thinner films also consist of smaller particles, which is in accordance with expectations. UV–vis spectra depicted in Figure 5 show that for a given centrifugation rate the optical transmittance of the deposited graphene film has a value that scales with the quantity of solvent used. The results thus indicate that dilution of solution-processed graphene can be used as a tool to control graphene film thickness. Also, the centrifugation rate can be controlled to make films of desired thickness.

Regardless of how the film thickness is controlled, an analysis of Figure 6 reveals that there are two different regimes, that is, one in which the optical transmittance sharply rises with increasing resistance, at lower transmittance values, and another in which the optical transmittance rises with increasing resistance to a much smaller extent, at higher transmittance values. It has been noted before that thin transparent conductors [45], including graphene [46], exhibit a percolation threshold. For film thicknesses above this threshold, the film behaves as a bulk material and the transmittance and sheet resistance *R*_S_ obey the following equation:


[1] $$T={\left[1+\frac{Z_{0}}{2R_{\text{S}}}\frac{\sigma_{op}}{\sigma_{dc,B}}\right]}^{-2}$$

where *Z*_0_ is the impedance of free space, σ_op_ is the optical conductivity, and σ_dc,B_ is the bulk dc conductivity of the film. For film thicknesses below the percolation threshold, the transmittance and sheet resistance obey the following equation:


[2] $$T={\left[1+\frac{1}{\Pi }{\left(\frac{Z_{0}}{R_{\text{S}}}\right)}^{1/\left(n+1\right)}\right]}^{-2}$$

where Π is the percolative figure of merit (FOM) as described by De and Coleman [45] and *n* is the percolation exponent.

We fit the above two equations to our data in the two observed regimes independently. The solid red line in Figure 6 is a fit to Equation 1 which indicates the bulk regime, whereas the dashed black line is a fit to Equation 2 for the percolation regime. The fit results in Π = 1.1 and *n* = 3.6, values which are of the same order as values previously observed for graphene [45]. The good match between our results and previously reported ones is yet another indication that the method that we used yields films of similar quality as observed earlier with other methods, although our method is facile and starts from commercially available material.

The two regimes exhibit a crossover that likely indicates the percolation threshold. The value of optical transparency at which we observe the threshold is 82.4%, matching the value found in an earlier work that showed particle size selection by control of liquid-phase exfoliation time [27], possibly indicating an upper limit for optical transparency achievable with Langmuir–Blodgett graphene films. The observed critical threshold transparency is equivalent to films which have a thickness of 7.6 layers of graphene, which is 2.6 nm. It is likely that reliably conducting films thinner than this cannot be achieved with Langmuir–Blodgett assembly of graphene nanoplatelets. Compared to the work in which the exfoliation time is controlled in the same lab where film deposition is made, our approach is advantageous because it allows experimenters to focus on film formation alone, leaving exfoliation to a partner lab or commercial vendor.

## Conclusion

Solution-processed graphene holds potential for applications across a diverse range of industries. There exist several production methods for solution-processed graphene, some of which are highly scalable. However, all graphene solutions resulting from those processes are polydisperse, containing a wide distribution of particle sizes, which is unfavorable for applications. It was previously shown that cascade centrifugation can be used as a common method for the separation of graphene particles by size, in the case of ultrasound-assisted liquid-phase exfoliated graphene. In this paper, we demonstrated that cascade centrifugation of electrochemically exfoliated graphene can be used in conjunction with Langmuir–Blodgett assembly to produce thin graphene films. By tuning centrifugation speed and solvent dilution volume, we produced films of different controlled thicknesses. We have shown that the optical transparency and electrical resistance of these films behave similarly to those of other films made of graphene from solution. Namely, the films exhibit a percolation threshold, at which optoelectronic properties experience a critical change. The percolation exponent and percolative FOM are found to be of the same order as in the case of other graphene films found in literature, which indicates that our films are of a similar quality. However, the method that we have used, which relies on cascade centrifugation of a commercially available solution of graphene, is easily accessible to researchers who have no exfoliation experience or equipment. We have shown that ownership of a centrifuge is the only prerequisite for making practical use of widely available graphene in solution.
